# Retrospective Review of a Prothrombin Complex Concentrate (Beriplex P/N) for the Management of Perioperative Bleeding Unrelated to Oral Anticoagulation

**DOI:** 10.1177/1076029617753537

**Published:** 2018-02-07

**Authors:** Pratima Chowdary, Augustine Tang, Dale Watson, Martin Besser, Peter Collins, Michael Desmond Creagh, Hafiz Qureshi, Margaret Rokicka, Tim Nokes, Paul Diprose, Ravi Gill

**Affiliations:** 1 Katharine Dormandy Haemophilia Centre and Thrombosis Unit, Royal Free Hospital, London, United Kingdom; 2 Lancashire Cardiac Centre, Blackpool Teaching Hospital FT, Blackpool, United Kingdom; 3 Faculty of Health and Medicine, Lancaster University, Lancaster, United Kingdom; 4 Addenbrooke’s Hospital, University of Cambridge, Cambridge, United Kingdom; 5 Arthur Bloom Haemophilia Centre, University Hospital of Wales, Cardiff, United Kingdom; 6 Department of Haematology, Royal Cornwall Hospital, Truro, United Kingdom; 7 Department of Transfusion Medicine, Glenfield Hospital, Leicester, United Kingdom; 8 Royal Blackburn Hospital, Blackburn, United Kingdom; 9 Derriford Hospital, Plymouth, United Kingdom; 10 Department of Anaesthesia, University Hospital Southampton, Southampton, United Kingdom; † Deceased

**Keywords:** bleeding, cardiovascular surgery, fresh frozen plasma, prophylaxis, prothrombin complex concentrate

## Abstract

A multicenter, retrospective, observational study of 4-factor prothrombin complex concentrate (PCC) and/or fresh frozen plasma (FFP) use within routine clinical care unrelated to vitamin K antagonists was conducted. The PCC was administered preprocedure for correction of coagulopathy (prophylactic cohort) and treatment of bleeding postsurgery (treatment cohort). Of the 445 patients included, 40 were in the prophylactic cohort (PCC alone [n = 16], PCC and FFP [n = 5], FFP alone [n = 19]) and 405 were in the treatment cohort (PCC alone [n = 228], PCC and FFP [n = 123], FFP alone [n = 54]). Cardiovascular surgery was the most common setting. PCC doses ranged between 500 and 5000 IU. Effectiveness (assessed retrospectively) was reported as effective in 93.0% in the PCC-only group (95% confidence interval, 89.1% to 95.9%), 78.9% (70.8% to 85.6%) with PCC and FFP, and 86.3% (76.2% to 93.2%) with FFP alone. In the treatment cohort, international normalized ratio was significantly reduced in all 3 groups. In patients who received PCC, the rate of thromboembolic events (1.9%) was below rates in the literature for similar procedures. PCCs offer a potential alternative to FFP in the management of perioperative bleeding unrelated to oral anticoagulant therapy.

## Background

Acquired coagulopathy is the most common indication for transfusing fresh frozen plasma (FFP).^1^ The aim of the treatment is to prevent procedure-related bleeding or manage acute bleeding by restoring deficient coagulation factors. A prolonged prothrombin time (PT) or a high international normalized ratio (INR) is often used as a trigger for transfusion,^2^ although neither PT nor INR is validated to predict risk of bleeding.^1,3^ Studies have shown that increments in coagulation factor levels following standard doses of FFP do not reliably correct coagulopathy, whereas prothrombin complex concentrates (PCCs; 20-50 IU/kg) or higher doses of FFP (∼30 mL/kg) can increase coagulation factor concentration to levels considered to be adequate for hemostasis.^4,5^

PCCs are well established for emergency reversal of oral anticoagulation with vitamin K antagonists.^6^ Beriplex P/N (CSL Behring, Marburg, Germany) is a 4-factor PCC containing coagulation factors II, VII, IX, and X along with proteins C, S, and Z. It is licensed in the United Kingdom and Europe for treatment and perioperative prophylaxis of bleeding related to acquired deficiencies in vitamin K-dependent coagulation factors, and for treatment and perioperative prophylaxis of bleeding in congenital deficiency of any of the vitamin K-dependent coagulation factors.^7^ In other countries such as the United States, PCCs are not licensed for the treatment of acquired coagulopathy. However, there has been increasing interest in using PCCs to treat bleeding beyond the setting of anticoagulation reversal, as they enable rapid administration of adequate doses of coagulation factors with small administration volumes. Unlike FFP, PCCs do not need to be thawed before administration, and they can be reconstituted rapidly.^8^ Furthermore, the manufacturing process for coagulation factor concentrates includes purification and viral inactivation, minimizing potential risks such as pathogen transmission, and transfusion-related acute lung injury.^9–12^

Limited retrospective studies have shown the effectiveness of PCCs in surgical bleeding.^13–17^ Recent European anesthesiology guidelines state that goal-directed therapy with coagulation factor concentrates (fibrinogen and/or PCC) may reduce transfusion-associated costs in trauma, cardiac surgery, and liver transplantation.^18^ American anesthesiology guidelines suggest PCCs for bleeding in the presence of elevated INR,^19^ and European trauma guidelines suggest administration of PCC to bleeding patients with thromboelastometric evidence of delayed coagulation initiation.^20^ However, because the published literature on this is limited, the primary basis for these recommendations is expert opinion. Relevant guidelines from the United Kingdom do not include recommendations for using PCC except for anticoagulation reversal.^21^

A multicenter, retrospective, observational study of 4-factor PCC use within routine clinical care in the United Kingdom was undertaken (“ProBe” study: *Pro*thrombin complex concentrate and *Be*riplex). The primary objective was to evaluate the clinical effectiveness of PCC for prophylaxis or treatment of perioperative bleeding in patients not taking oral anticoagulant therapy (ie, vitamin K antagonists). Secondary objectives were to assess safety and tolerability of PCC by frequency of adverse drug reactions (ADRs); to evaluate the dose–response relationship with PCC; and to compare characteristics of patients treated with PCC with those of FFP-treated patients.

## Methods

### Study Design

This was a multicenter, retrospective, noninterventional, observational study of 4-factor PCC, conducted in 9 centers in the United Kingdom over a period of 20 months (May 2010-January 2012). The study was carried out in accordance with the International Conference on Harmonization Good Clinical Practice guidelines and the Declaration of Helsinki and registered at www.clinicaltrials.gov (NCT01053169). The study was approved by an independent ethics committee (National Hospital for Neurology and Neurosurgery and Institute of Neurology Research Ethics Committee—09/H0716/74), and informed consent was waived for the data collection. The waiver was provided because PCC was often a small component of patients’ overall treatment (patients were unlikely to be familiar with every intervention), and to ensure data validity, there was a need to include all potentially eligible patients (not all patients were available for discussion and consent postintervention).^22^ The study was funded and sponsored by CSL Behring.

Standard practices in the participating centers were followed for the administration of PCC and FFP and sampling for assessment of hemostasis. Based on the reason for treatment with PCC and/or FFP, 2 patient cohorts were defined: a prophylactic cohort and a treatment cohort. The prophylactic cohort included patients with acquired coagulopathy presenting with prolonged PT or INR due to a medical condition who required surgical or diagnostic intervention and received PCC and/or FFP to prevent bleeding. Patients in the treatment cohort received PCC and/or FFP to stop or reduce acute perioperative bleeding.

### Patient Cohort and Treatment Details

Patients who received Beriplex P/N and/or FFP for prevention or treatment of bleeding were potentially eligible for inclusion. The decision to use PCC was dictated by individual clinicians based on institutional practice. Other inclusion criteria for both cohorts were age ≥16 years, administration of FFP or PCC, and availability of the following information: reason for treatment with PCC and/or FFP, administered dose, treatment outcome, blood product transfusion, and concurrent hemostatic therapy given. Additional inclusion criteria for the prophylactic cohort were coagulopathy, defined as INR >1.4 or PT ≥3 seconds above the upper limit of the reporting laboratory’s normal range and any planned major or minimally invasive procedure. The exclusion criteria for both cohorts were treatment with PCC other than Beriplex P/N and reversal of anticoagulation secondary to oral vitamin K antagonist therapy.

Centers were encouraged to include all potentially eligible patients who received PCCs between May 2010 and January 2012. A small group of patients who received FFP alone for the same reasons was also included to observe standard practice, and recruitment to this arm was by competitive enrollment until target numbers were reached.

For each study participant, the following data were entered into an electronic database: patient characteristics, relevant medical history, vital signs, diagnosis, intervention undertaken, cause of coagulopathy, source of bleeding, and reason(s) for administering PCC or FFP (ie, all clinical triggers for treatment). The clinical triggers for administering PCCs included blood loss (eg, >20%), ongoing microvascular (nonsurgical) bleeding as assessed by the surgeon, abnormal coagulation parameters (eg, INR and PT), abnormalities on point-of-care testing; clinicians were asked to indicate all relevant indications for each patient. In addition, details of treatment with other blood products or hemostatic therapy including red blood cells, platelets, cryoprecipitate, fibrinogen concentrate, vitamin K, desmopressin, tranexamic acid, and recombinant activated factor VII were collected. When available, INR and other laboratory parameters (eg, activated partial thromboplastin time, hemoglobin, platelet count, and fibrinogen concentration) were documented at 3 time points: before treatment and at about 3 and 24 hours posttreatment. Both surgical and microvascular bleeding were included.

The clinical effect of PCC/FFP was assessed retrospectively by the investigators, based on patient notes at each center. Perceived effectiveness in preventing or stopping bleeding was rated as “very good” (prompt cessation of bleeding and/or rapid fall in INR [<1.6]; hemostasis during surgery not significantly different from normal), “satisfactory” (delayed cessation of bleeding and/or delayed fall in INR [1-2 hours]; mildly abnormal hemostasis during surgery in terms of quantity and/or quality [slight oozing]), “questionable,” or “doubtful” (cessation of bleeding and fall in INR after >2 hours; moderately abnormal hemostasis during surgery [controllable bleeding]), “none” (lack of effect on bleeding and insufficient fall in INR; severely abnormal hemostasis during surgery [severe hemorrhage, difficult to control]), or “no judgment possible.” The definitions for each category were subjective and based on those utilized in prior clinical trials of PCC and surgery in patients with hemophilia.^23,24^ One overall rating was provided for each patient regardless of the number of doses of PCC and/or FFP administered. Adequate effectiveness was defined as very good or satisfactory effectiveness in preventing/stopping bleeding. The effect of PCCs on INR was also assessed.

The ADRs that were judged by the investigator to be at least possibly related to PCC (including thromboembolic events), and all deaths among recipients of PCC (irrespective of cause), were collected during a 24-hour observation period following the last administration of PCC. Neither ADRs nor deaths were collected in relation to FFP.

### Statistical Analysis

A sample size calculation was not performed because of the observational nature of the study and the lack of previous data. A minimum total of 200 patients was planned to obtain sufficient evaluable patients treated with PCC. For the group treated with FFP alone, a maximum of 50 patients was allowed to be documented. After database lock, it was decided that a regrouping of the patients would be useful to analyze the data, with 3 groups described within each cohort: PCC alone, FFP alone, and PCC and FFP. No statistical comparisons were made between these groups.

Clinical effectiveness was assessed by descriptive statistics in keeping with the primary and secondary objectives. The treatment was considered effective when a rating of very good or satisfactory was provided by the investigator. Results are presented as mean and standard deviation for parameters following the normal distribution and median and interquartile range (IQR) for nonnormal distributions.

The relationship between total PCC dose and clinical effectiveness was analyzed by categorizing patients into dose groups (for patients with more than 1 administration of PCC, the total dose was calculated from all administrations). Three dose categories were defined (<10 IU/kg, 10-19.9 IU/kg, and ≥20 IU/kg, corresponding approximately to doses of 500, 1000, and ≥1500 IU, respectively). Responses of “no judgment possible” were considered to be missing data, and these patients were excluded. Fisher’s exact test was used to examine whether there was an association between dose and a number of categorical variables, including the percentage of patients in whom PCC was deemed to have adequate effectiveness. The Kruskal-Wallis test was used to compare continuous variables between the 3 dose categories. The criterion for statistical significance was set as *P* < .05. As concurrent transfusion of other hemostatic products is a major confounder, details of blood product usage stratified by PCC dose are provided.

## Results

### Patient Characteristics

A total of 445 patients were recruited: 40 in the prophylactic cohort and 405 in the treatment cohort. In total, 372 patients received PCC with or without FFP and 73 received FFP alone. Patient characteristics (Table 1) were generally consistent across patients receiving FFP alone, PCC alone, or PCC and FFP (treatment and prophylactic cohorts combined). However, platelet count and hemoglobin were numerically lower among patients receiving PCC and FFP versus those receiving either treatment alone. No ADR data were available for 4 recipients of PCC who were consequently excluded from the safety population (n = 368).

**Table 1.** Baseline Characteristics of Patients.

	By Cohort		By Type of Treatment Administered			
	Prophylactic Cohort, n = 40	Treatment Cohort, n = 405	PCC Alone, n = 244	PCC and FFP, n = 128	FFP Alone, n = 73	All Patients, N = 445
Gender, n (%), N = 445						
Male	20 (50.0)	259 (64.0)	149 (61.1)	84 (65.6)	46 (63.0)	279 (62.7)
Female	20 (50.0)	146 (36.0)	95 (38.9)	44 (34.4)	27 (37.0)	166 (37.3)
Age, years, N = 445						
Mean (SD)	59.0 (18.7)	69.6 (13.3)	69.4 (13.9)	66.9 (15.8)	69.1 (12.2)	68.6 (14.2)
Height, cm, n = 418						
Mean (SD)	166.7 (7.8)	168.9 (10.9)	168.2 (11.4)	169.7 (10.3)	168.6 (9.0)	168.7 (10.7)
Weight, kg, N = 445						
Mean (SD)	73.1 (18.8)	77.1 (15.6)	75.9 (16.0)	78.8 (16.8)	75.8 (14.3)	76.7 (16.0)
Body mass index, kg/m^2^, n = 418						
Mean (SD)	26.5 (7.0)	27.0 (4.8)	26.8 (5.0)	27.6 (5.2)	26.8 (4.5)	27.0 (5.0)
Hemoglobin, g/dL^a^, n = 307						
Mean (SD)	9.7 (2.5)	9.4 (1.9)	9.6 (1.8)	9.1 (1.9)	9.9 (2.7)	9.5 (2.0)
Platelet count, ×10^9^/L^a^, n = 248						
Median (IQR)	176 (65, 267)	130 (98, 164)	136 (102, 185)	115 (86, 161)	134 (106, 198)	130 (96, 171)

Abbreviations: FFP, fresh frozen plasma; IQR, interquartile range; PCC, prothrombin complex concentrate; SD, standard deviation

^a^Measurement taken within 3 hours before study medication.

In 93% of prophylactic cohort (37/40 patients), an abnormal coagulation test result prior to a surgical procedure was the indication for treatment. The remaining 3 (8%) patients had a history of excessive bleeding. Reasons for PCC administration in the treatment cohort, in addition to bleeding, were documented as follows: 43% (175/405 patients) had abnormal point-of-care test results and abnormal laboratory test results were seen in 27% (109/405 patients). Microvascular bleeding was noted in 23% of the treatment cohort (93/405 patients), and no coagulation test result was available in 41% (166/405 patients). These patients were treated for diffuse oozing and clinically diagnosed bleeding. Details of the patients’ interventions and/or procedures are shown in Table 2. Cardiovascular surgery (eg, coronary artery bypass graft [CABG]) was the most common setting in the treatment cohort, and this is a reflection of the centers participating in the study.

**Table 2.** Interventions/Procedures Performed in (A) the Prophylactic and (B) the Treatment Cohort, by Treatment Administered.

(A) Prophylactic Cohort	PCC Alone, n = 16	PCC and FFP, n = 5	FFP Alone, n = 19	All Patients, n = 40
Intervention/procedure				
Minor procedure	8	3	11	22
Lumbar puncture or epidural	3	0	3	6
Central venous lines–placement and removal	0	2	0	2
Arterial access	2	1	0	3
Tracheostomy	0	0	2	2
Liver biopsy	0	0	2	2
Endoscopy with/without endoscopic retrograde cholangiopancreatography (ERCP)	0	0	3	3
Chest drain with pleural aspiration	1	0	0	1
Other (including patients undergoing multiple procedures)	2	0	1	3
Major procedure	8	2	8	18
Abdominal surgery	2	0	5	7
Cranial surgery	5	1	0	6
Major vascular surgery	0	1	2	3
Other	1	0	1	2
(B) Treatment Cohort				
Intervention/procedure	PCC Alone, n = 228	PCC and FFP, n = 123	FFP Alone, n = 54	All Patients, n = 405
Cardiovascular surgery	218	113	51	382
Isolated first-time CABG	60	22	23	105
Isolated aortic valve replacement	30	9	10	49
Mitral valve replacement or repair with/without tricuspid valve replacement	26	13	2	41
Aortic valve replacement with CABG	28	12	8	48
Mitral valve replacement or repair (± tricuspid valve replacement) with CABG	22	3	2	27
Multiple valve procedure with/without CABG	11	12	1	24
Simple aortic surgery	16	13	2	31
Complex aortic surgery	4	4	0	8
Other complex surgery (including endocarditis and re-do surgery)	21	25	3	49
Gastrointestinal surgery	0	7	2	9
Neurological/nervous system surgery	2	2	0	4
Musculoskeletal surgery	1	0	0	1
Other surgical procedures	7	1	1	9

Abbreviations: CABG, coronary artery bypass graft; FFP, fresh frozen plasma; PCC, prothrombin complex concentrate

### Treatment Details Including Allogeneic Blood Products

The median total dose of PCC in the prophylactic cohort was 1500 IU (22.5 IU/kg; 60 mL; IQR, 1250-2125 IU), and in the treatment cohort, it was 1000 IU (11.5 IU/kg; 40 mL; IQR, 500-1000 IU). Most patients received only 1 dose of PCC: 85.7% (18/21 patients) in the prophylactic cohort and 87.2% (306/351 patients) in the treatment cohort. In the prophylactic cohort, the remaining 3 patients received 2 doses, while in the treatment cohort 10.5% (37/351 patients) received 2 doses and 2.3% (8/351 patients) received 3 doses. Single doses ranged between 500 and 5000 IU.

The median total dose of FFP among recipients of FFP-only in the prophylactic cohort was 1000 mL (14.5 mL/kg; IQR, 1000-1000 mL), and in the treatment cohort, it was 825 mL (11.0 mL/kg; IQR, 550-1100 mL). Similar to PCC, 94.7% (18/19 patients) of the prophylactic cohort treated with FFP alone received only 1 dose, compared to 87.0% (47/54 patients) in the treatment cohort. In the prophylactic cohort, 1 (5.3%) patient received 2 doses, while in the treatment cohort 3 (5.6%) patients received 2 doses, 2 (3.7%) patients received 3 doses, and 2 (3.7%) patients received 4 doses.

Platelets were the most commonly administered blood product after PCC/FFP, with 35.0% of patients in the prophylactic cohort and 60.0% of those in the treatment cohort receiving this treatment. The second and third most commonly administered products were red blood cells (prophylactic cohort, 37.5%; treatment cohort, 46.4%) and cryoprecipitate (2.5%; 25.2%). Tranexamic acid was administered to 10.0% of patients in the prophylactic cohort and 1.5% of those in the treatment cohort. Recombinant activated factor VII was administered to 5.0% of patients in the prophylactic cohort and 2.2% of those in the treatment cohort, and fibrinogen concentrate was given to 10.0% and 1.0%, respectively.

### Response to Treatment: Clinical Effectiveness, Transfusion, and Laboratory Parameters

**Figure 1.**
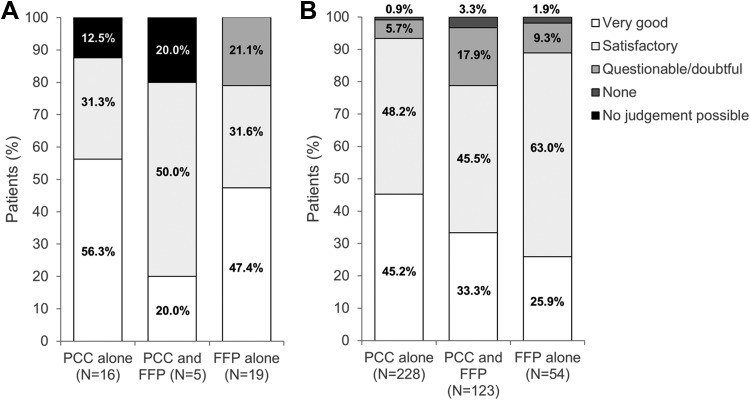
Effectiveness in (A) the prophylactic cohort and (B) the treatment cohort. Effectiveness was judged by the physician as very good, satisfactory, questionable/doubtful, none, or no judgment possible with regard to preventing bleeding (prophylactic cohort) or stopping bleeding (treatment cohort). FFP indicates fresh frozen plasma; PCC, prothrombin complex concentrate.

Across both cohorts (prophylactic and treatment), treatment was reported to be effective in 93.0% of patients receiving PCC alone (95% confidence interval [CI]: 89.1% to 95.9%), in 78.9% (70.8% to 85.6%) of those receiving PCC and FFP, and in 86.3% (76.2% to 93.2%) of patients receiving FFP alone. When analyzed separately, in the prophylactic cohort, treatment was reported to be effective in 87.5% of patients who received PCC alone, 80.0% of patients who received PCC and FFP, and 78.9% of recipients of FFP alone (Figure 1). Corresponding percentages in the treatment cohort were 93.4%, 78.9%, and 88.9%, respectively.

Statistical analysis of the dose–response relationship was restricted to the treatment cohort, as there were too few patients in the prophylactic cohort for meaningful comparisons. No significant association was noted between dose and effectiveness among patients receiving PCC alone, while the incidence of adequate effectiveness decreased significantly with increasing PCC dose among patients receiving PCC and FFP (Table 3). Increasing doses of PCC were associated with significantly higher utilization of blood product transfusion, and this was reflected in the data for individual blood components (red blood cells, platelets, FFP, and cryoprecipitate). Consequently, treatment cohort patients who received PCC and FFP with a PCC dose of ≥20 IU/kg had the lowest reported effectiveness and the highest utilization of blood products (including red blood cells), indicating combined use of multiple treatments in the management of excessive bleeding. In the prophylactic cohort (patients receiving PCC alone), there was a nonsignificant trend toward a lower incidence of blood product transfusion with a PCC dose of ≥20 IU/kg compared to doses of 10.0 to 19.9 IU/kg.

**Table 3.** Effectiveness and Interventions According to Treatment and PCC Dose: (A) Prophylactic Cohort and (B) Treatment Cohort.

(A) Prophylactic Cohort								
Parameter	PCC Dose (Recipients of PCC Only)^a^			*P* value^b^	PCC Dose (Recipients of PCC and FFP)			*P* value^b^
	10-19.9 IU/kg	≥20 IU/kg			10-19.9 IU/kg (Median FFP dose, 1093 mL)	≥20 IU/kg (Median FFP dose, 1625 mL)		
Number of patients	7	9			1	4		
Baseline INR, median (IQR)	1.6 (1.4,1.7)	1.9 (1.8, 1.9)		.13	2.1 (2.1, 2.1)	2.3 (1.8, 3.0)		NC
Adequate Effectiveness^c^	7 (100%)	7 (100%)^d^		1.00	-^d^	4 (100%)		NC
Any blood product transfusion	4 (57%)	4 (44%)		1.00	1 (100%)	4 (100%)		NC
Platelets								
Recipients, n (%)	4 (57%)	2 (22%)		.30	1 (100%)	2 (50%)		NC
Median dose (IQR)	1 (0, 2)	0 (0, 0)		.14	4 (4, 4)	1 (0, 3)		NC
Red blood cells								
Recipients, n (%)	1 (14%)	2 (22%)		1.00	1 (100%)	3 (75%)		NC
Median dose (IQR)	0 (0, 0)	0 (0, 0)		.82	3 (3, 3)	2 (1, 4)		NC
FFP								
Recipients, n (%)	0 (0%)	0 (0%)		1.00	1 (100%)	4 (100%)		NC
Median dose (IQR)	0 (0, 0)	0 (0, 0)		1.00	4.0 (4.0, 4.0)	5.9 (3.8, 8.7)		NC
Cryoprecipitate								
Recipients, n (%)	0 (0%)	0 (0%)		1.00	0 (0%)	0 (0%)		NC
Median dose (IQR)	0 (0, 0)	0 (0, 0)		1.00	0 (0, 0)	0 (0, 0)		NC
(B) Treatment Cohort								
	PCC Dose (Recipients of PCC Only)			*P* Value^b^	PCC dose (Recipients of PCC and FFP)			*P* Value^b^
Parameter	<10 IU/kg	10-19.9 IU/kg	≥20 IU/kg		<10 IU/kg (Median FFP dose, 550 mL)	10-19.9 IU/kg (Median FFP dose, 544 mL)	≥20 IU/kg (Median FFP dose, 560 mL)	
Number of patients	113	89	26		32	59	32	
Baseline INR, median (IQR)	1.5 (1.4, 1.7)	1.5 (1.4, 1.7)	1.5 (1.4, 1.7)	.33	1.5 (1.4, 1.7)	1.7 (1.5, 1.9)	1.4 (1.2, 2.4)	.12
Adequate Effectiveness^c^	107 (95%)	83 (93%)	23 (88%)	.48	26 (81%)	51 (86%)	20 (63%)	.03
Any blood product transfusion	50 (45%)	58 (67%)	22 (88%)	<.001	21 (72%)	51 (93%)	30 (94%)	.03
Platelets								
Recipients, n (%)	39 (35%)	50 (56%)	20 (77%)	<.001	21 (70%)	51 (88%)	30 (94%)	.03
Median dose (IQR)	0 (0, 1)	1 (0, 2)	1 (1, 2)	<.001	2 (0, 2)	2 (1, 2)	3 (2, 3)	<.001
Red blood cells								
Recipients, n (%)	33 (29%)	40 (45%)	13 (52%)	.02	14 (45%)	40 (68%)	25 (78%)	.02
Median dose (IQR)	0 (0, 1)	0 (0, 2)	1 (0, 3)	.02	0 (0, 2)	2 (0, 4)	2 (1, 7)	.004
FFP								
Recipients, n (%)	0 (0%)	0 (0%)	0 (0%)	1.00	32 (100%)	59 (100%)	32 (100%)	1.00
Median dose (IQR)	0 (0, 0)	0 (0, 0)	0 (0, 0)	1.00	2.0 (1.4, 2.4)	2.0 (1.9, 2.9)	2.0 (2.0, 4.0)	.02
Cryoprecipitate								
Recipients, n (%)	5 (4%)	15 (17%)	13 (50%)	<.001	5 (16%)	21 (38%)	22 (69%)	<.001
Median dose (IQR)	0 (0, 0)	0 (0, 0)	0.5 (0, 2)	<.001	0 (0, 0)	0 (0, 1)	2 (0, 2)	<.001

Abbreviations: FFP, fresh frozen plasma; IQR, interquartile range; NC, not calculated; PCC, prothrombin complex concentrate.

^a^In the prophylactic cohort, no patients received PCC at a dose below 10 IU/kg.

^b^ *P* values are for differences between dose groups.

^c^Adequate effectiveness defined as very good or satisfactory effectiveness in prevention/stopping of bleeding (physician’s judgment).

^d^Two missing values ≥20 IU/kg group for PCC only patients, and 1 missing value for 10 to 19.9 IU/kg for PCC and FFP patients.

Baseline INR values and responses to treatment are shown in Table 4. Decreases in median values were observed among recipients of PCC and/or FFP, in both cohorts, between baseline and 3 hours postdose. Little change was observed between 3 and 24 hours postdose. Where data were available at both baseline and 3 hours postdose, statistically significant improvements in INR were evident in most of the patient groups.

**Table 4.** International Normalized Ratio (INR) Results.

	All Available Data^a^			3 Hours Postdose vs Baseline–Data Only for Patients With Values at Both Time Points	
Patients	Baseline	3 Hours Postdose	24 Hours Postdose	Change, Median (95% CI)	*P* Value
INR: Prophylactic cohort					
Patients receiving PCC alone (n = 16)	1.7 (1.5-1.9), n = 14	1.4 (1.2-1.5), n = 10	1.5 (1.4-1.6), n = 11	−.3 (−.4 to −0.1) n = 10	.005
Patients receiving PCC & FFP (n = 5)	2.1 (1.8-3.0), n = 5	1.8 (1.6-2.4), n = 5	1.9 (1.6-2.2), n = 5	−.4 (−.8 to 0), n = 5	.06
Patients receiving FFP alone (n = 19)	1.7 (1.6-1.9), n = 13	1.4 (1.3-1.5), n = 15	1.4 (1.3-1.5), n = 11	−.3 (−.6 to −.2), n = 13	.001
INR: Treatment cohort					
Patients receiving PCC alone (n = 228)	1.5 (1.3-1.7), n = 131	1.2 (1.2-1.2), n = 176	1.2 (1.1-1.3), n = 124	−.3 (−.4 to−.2), n = 96	<.001
Patients receiving PCC & FFP (n = 123)	1.6 (1.4-1.9) n = 62	1.2 (1.1-1.4), n = 90	1.2 (1.1-1.4) , n = 39	−.4 (−.5 to −.3), n = 49	<.001
Patients receiving FFP alone (n = 54)	1.4 (1.2–1.6), n = 27	1.2 (1.1-1.3), n = 36	1.2 (1.0-1.2), n = 40	−.3 (−.5 to .1), n = 14	.002

Abbreviations: CI, confidence interval; FFP, fresh frozen plasma; PCC, prothrombin complex concentrate.

^a^Data shown are median (interquartile range).

### Safety and Tolerability (Data Collected Only for Patients Receiving PCC)

The ADRs reported could be broadly categorized either as ineffective therapy or as thromboembolic events (Table 5). “Drug ineffective” was reported in 6 treatment cohort patients (1.6% of all PCC recipients).

**Table 5.** Safety Outcomes Among all Recipients of Prothrombin Complex Concentrate (PCC): Numbers of Affected Patients (%).

Event	Prophylactic Cohort (n = 21)	Treatment Cohort (n = 347)	Both Cohorts Combined (n = 368)
Any adverse drug reaction^a^	1 (4.8)^b^	6 (1.7)^c^	7 (1.9)
Drug ineffective	0 (0.0)	6 (1.7)	6 (1.6)
Hemorrhage	0 (0.0)	3 (.9)	3 (.8)
Thromboembolic event	1 (4.8)	6 (1.7)	7 (1.9)^d^
Cerebral ischemic event	0 (0.0)	5 (1.4)	5 (1.4)
Myocardial infarction	1 (4.8)	1 (.3)	2 (.5)
Death	3 (14.3)	9 (2.6)	12 (3.3)

^a^Excluding thromboembolic events and deaths.

^b^One patient had relapsed acute promyelocytic leukemia and acute myocardial infarction

^c^Details of the adverse drug reactions in these 6 patients are as follows: “drug ineffective” (3 patients); “drug ineffective” and hemorrhage (2 patients); and atrial fibrillation, atrial flutter, cerebral infarction, cerebrovascular accident, delayed recovery from anesthesia, disturbance in attention, “drug ineffective,” hemorrhage, hemiparesis, mechanical ventilation, nervous system disorder, pericardial hemorrhage, retinal artery embolism, tracheostomy, unresponsive to stimuli, ventricular arrhythmia, vision blurred, and visual acuity reduced (1 patient)

^d^Additional treatment was administered to these patients as follows: platelet concentrate (n = 7), vitamin K (n = 6), desmopressin (n = 6), cryoprecipitate (n = 6), red blood cells (n = 5) and fibrinogen (n = 1; see Supplementary Table 1).

Thromboembolic events occurred in 7 (1.9%) PCC recipients during the 24-hour observation period, and all except 1 were in the treatment cohort. All of these events were arterial and 1 was associated with patient death (see below). All were considered by the investigators to be possibly related to PCC therapy. Additional treatments received by patients with thromboembolic events (most commonly platelet concentrate [n = 7], vitamin K [n = 6], desmopressin [n = 6], cryoprecipitate [n = 6], and red blood cells [n = 5]) are shown in Supplementary Table 1.

Twelve deaths occurred among PCC recipients during the 24-hour observation period, and one of these was assessed by the investigator to have been possibly related to PCC treatment. The patient, a 70-year-old woman in the prophylactic cohort with a diagnosis of acute promyelocytic leukemia, was treated with 2 doses of PCC on consecutive days (1750 IU [27 IU/kg] and 1000 IU [16 IU/kg]). Two units of platelet concentrate were also administered. Death occurred on the same day that the second dose of PCC was given. The cause of death was reported as “relapsed acute promyelocytic leukemia and acute myocardial infarction.” The patient was being treated with all-trans retinoic acid, but her history in relation to ischemic heart disease is unavailable. Causes of death for the remaining 11 patients were listed as follows: multiorgan failure secondary to overwhelming sepsis; neutropenic sepsis, multiorgan failure; end-stage renal disease, diabetes mellitus, multiorgan failure; advanced lung cancer, low vital capacity; acute hemorrhagic pancreatitis; multiple organ failure due to sepsis, endocarditis, and pneumonia; heart failure; ischemic bowel disease, accelerated ventricular rhythm; ruptured hepatic artery aneurysm; multiorgan failure, tricuspid valve disease, mitral valve disease, chronic obstructive pulmonary disease; and bowel infarction, myocardial infarction, renal and hepatic failure, and ischemic heart disease (operated).

Three additional deaths occurred outside the 24-hour observational period, all in the treatment cohort patients. All three patients had a thromboembolic adverse event: 2 cerebral ischemic events and 1 myocardial infarction. The lengths of time between study drug administration and death were 4 days, 114 days, and 188 days. One of these deaths (the patient with myocardial infarction who died 4 days after PCC therapy) was considered by the investigator to be possibly related to study drug.

## Discussion

To date, this is the largest retrospective study on the clinical use of PCCs for acquired coagulopathy and/or perioperative bleeding in routine practice. The results show that PCC was used across a variety of clinical settings with wide dose ranges. Its clinical effectiveness was judged as very good or satisfactory in 93.0% of patients receiving PCC alone (87.5% in the prophylactic cohort and 93.4% in the treatment cohort). The corresponding effectiveness rate among patients receiving FFP only was 86.3%, but this result cannot be used for comparison. The PCC produced statistically significant reductions in INR. For example, 3 hours after PCC infusion in the prophylactic cohort (PCC alone group), the mean INR was <1.5. The incidence of thromboembolic events with PCC was lower than in the published literature for the cohort under treatment.^25–28^

Around 90% of patients received only 1 dose of PCC. In the treatment cohort, there was potential to titrate the dose upward if required, while the intention in the prophylactic cohort was to administer a single, effective dose. In the treatment cohort, a wide range of PCC doses were used with no apparent association between dose and effectiveness with PCC alone. However, in the PCC and FFP group, PCC dose and effectiveness were inversely related. Patients receiving the highest PCC doses (≥20 IU/kg) had the highest utilization of blood products, suggesting selection bias toward more severely bleeding patients.

Published studies on the use of PCC for bleeding unrelated to oral anticoagulation in nonspecific or cardiac surgery settings are limited. In a small study of 16 patients with bleeding related to cardiac and noncardiac surgery, despite near-normal coagulation tests, PCC administration (average dose <1500 IU) was associated with partial or complete hemostasis in 75% of the patients.^14^ A retrospective study of 38 noncardiac surgery patients with severe perioperative bleeding reported that PCC (median dose 2000 IU) stopped bleeding in 96% of patients with microvascular bleeding and 36% of patients with surgical bleeding within 3 hours of administration.^16^ In another retrospective study in bleeding related to cardiopulmonary bypass, low-dose PCC (10-14 IU/kg) was associated with decreased postoperative bleeding.^13^ Similarly, 2000 IU PCC normalized PT and stopped bleeding in all 16 patients in a study of acquired coagulopathy in critical care.^29^ The doses of PCC in the present study are similar to or lower than those of the previous studies but lower than those used for reversal of oral anticoagulation (typically 25-50 IU/kg, depending on baseline INR^23,30^).

In our study, bleeding after cardiac surgery was the dominant setting for PCC use. Around 6% of patients bleed excessively after cardiac surgery, and this is a major cause of mortality and morbidity.^31^ Studies in patients with excessive blood loss after cardiac surgery have shown a variety of coagulation-related deficiencies including low platelet counts, low levels of coagulation factors IX, X, XI, and low endogenous thrombin potential.^32–34^ Thus, bleeding is multifactorial in nature, and a range of different hemostatic treatments may be needed.^18,34,35^ This is reflected in the current study where 60% of patients treated for perioperative bleeding received platelet concentrate, 46% received red blood cells, and 25% received cryoprecipitate, in addition to PCC and/or FFP. The PCC has been investigated within treatment algorithms as a second-line treatment for perioperative bleeding following fibrinogen supplementation.^35–37^

Based on published concentrations of coagulation factors within Beriplex P/N and FFP,^38^ the median PCC dose in this study resulted in the administration of more than 50% higher quantities of FII, FIX, and FX compared to the median dose of FFP.^9,39^ Several in vitro studies have demonstrated the influence of prothrombin and antithrombin levels on thrombin generation, with increasing levels of the former and decreasing levels of the latter associated with higher thrombin generation.^40–42^ Ex vivo spiking studies of plasma samples taken after cardiopulmonary bypass surgery have shown restoration of thrombin generation with PCCs.^43,44^

The potential for thrombogenicity is a key safety consideration when administering PCC.^45,46^ A recent pharmacovigilance study of Beriplex P/N, covering 647,250 applications over a 16-year period (mainly for reversal of vitamin K antagonists), reported that the incidence of thromboembolic events is ∼1:31,000 treatments.^47^ In this context, patients’ underlying conditions and the administration of other treatment inevitably affect the risk of thromboembolic adverse events. Reported incidences of deep vein thrombosis after cardiac surgery are as high as 15% to 46%, while pulmonary embolism occurs in 0.3% to 9.5% of patients.^28,48^ For CABG, 1% to 5% of patients are affected by stroke, and myocardial infarction has been reported to occur in 4% to 8% of patients.^25,26^ These percentages are higher than the 1.9% of PCC recipients in this study in whom thromboembolic events were reported, but we collected data only for the first 24 hours posttreatment, and this was a limitation of the study. Overall, in the present study, there were multiple risk factors for thromboembolic complications aside from the use of PCC.

This study has several additional limitations, including its retrospective nature, variations between centers in routine clinical practice, and the subjective method of assessing effectiveness. The criteria for administration of PCC and FFP (particularly in the prophylactic cohort) were related to practices of the individual clinicians and institutes included in the study; variability in this respect is reflected by the fact that 41% of the patients had no documented evidence of hemostatic impairment. Inappropriate use of FFP in surgery/trauma is well-documented, and the present study may have included patients not requiring hemostatic intervention.^49^ Furthermore, the use of tranexamic acid in the patients undergoing cardiovascular surgery was limited; strong evidence supporting the use of this drug has emerged since the study was performed.^50^ The retrospective nature of the study and the method for assessing effectiveness introduced potential for bias. However, the observation of similar effectiveness rates with either FFP or PCC could be interpreted as suggesting a similar impact of bias with both treatments. The study was not designed to compare the effectiveness of PCC versus FFP despite the inclusion of patients receiving both of these treatments. Numbers of patients receiving FFP alone and PCC alone were not balanced, and ratios of patient numbers across the study groups do not reflect those of clinical practice. Also, the safety analysis was limited by the collection of data only for patients who received PCC and only for a short period of time (24 hours) and by the lack of collection of safety data for FFP.

## Conclusions

In conclusion, these real-life data show that PCC can be effective in perioperative and intensive care as a single agent and as part of multifaceted hemostatic intervention in the management of perioperative bleeding. One advantage of using coagulation factor concentrates as opposed to allogeneic blood products is that they can be prepared and administered more quickly. Further studies with defined indications and more objective criteria for efficacy assessment are needed to ascertain the role of PCC in the management of perioperative bleeding.

## Supplemental Material

Supplemental Material, Probe_study_supplementary_information_20Jul2017 - Retrospective Review of a Prothrombin Complex Concentrate (Beriplex P/N) for the Management of Perioperative Bleeding Unrelated to Oral Anticoagulation Click here for additional data file. Supplemental Material, Probe_study_supplementary_information_20Jul2017 for Retrospective Review of a Prothrombin Complex Concentrate (Beriplex P/N) for the Management of Perioperative Bleeding Unrelated to Oral Anticoagulation by Pratima Chowdary, Augustine Tang, Dale Watson, Martin Besser, Peter Collins, Michael Desmond Creagh, Hafiz Qureshi, Margaret Rokicka, Tim Nokes, Paul Diprose, and Ravi Gill in Clinical and Applied Thrombosis/Hemostasis

## References

[1] Stanworth SJ Walsh TS Prescott RJ; Intensive Care Study of Coagulopathy (ISOC) investigators. A national study of plasma use in critical care: clinical indications, dose and effect on prothrombin time. Crit Care. 2011;15(2): R108. 21466676 10.1186/cc10129 PMC3219386

[2] Iorio A Basileo M Marchesini E The good use of plasma. A critical analysis of five international guidelines. Blood Transfus. 2008;6(1):18–24. 18661920 10.2450/2008.0041-07 PMC2626858

[3] Puetz J Fresh frozen plasma: the most commonly prescribed hemostatic agent. J Thromb Haemost. 2013;11(10):1794–1799. 23848285 10.1111/jth.12351

[4] Chowdary P Saayman AG Paulus U Findlay GP Collins PW Efficacy of standard dose and 30 ml/kg fresh frozen plasma in correcting laboratory parameters of haemostasis in critically ill patients. Br J Haematol. 2004;125(1):69–73. 15015971 10.1111/j.1365-2141.2004.04868.x

[5] Makris M Greaves M Phillips WS Kitchen S Rosendaal FR Preston EF Emergency oral anticoagulant reversal: the relative efficacy of infusions of fresh frozen plasma and clotting factor concentrate on correction of the coagulopathy. Thromb Haemost. 1997;77(3):477–480. 9065997

[6] Chai-Adisaksopha C Hillis C Siegal DM Prothrombin complex concentrates versus fresh frozen plasma for warfarin reversal. A systematic review and meta-analysis. Thromb Haemost. 2016;116(5):879–890. 27488143 10.1160/TH16-04-0266

[7] Electronic Medicines Compendium. Beriplex P/N 500 summary of product characteristics. https://www.medicines.org.uk/emc/medicine/20797. Published 2016. Accessed July 2017.

[8] Tanaka KA Szlam F Treatment of massive bleeding with prothrombin complex concentrate: argument for. J Thromb Haemost. 2010;8(12):2589–2591. 20831614 10.1111/j.1538-7836.2010.04052.x

[9] Scott LJ Prothrombin complex concentrate (Beriplex P/N). Drugs 2009;69(14):1977–1984. 19747012 10.2165/11203690-000000000-00000

[10] MacLennan S Williamson LM Risks of fresh frozen plasma and platelets. J Trauma. 2006;60(suppl 6):S46–SD50. 16763481 10.1097/01.ta.0000199546.22925.31

[11] Pandey S Vyas GN Adverse effects of plasma transfusion. Transfusion. 2012;52(suppl 1):65S–79S. 22578374 10.1111/j.1537-2995.2012.03663.x PMC3356109

[12] Franchini M Lippi G Prothrombin complex concentrates: an update. Blood Transfus. 2010;8(3):149–154. 20671873 10.2450/2010.0149-09 PMC2906185

[13] Arnekian V Camous J Fattal S Rézaiguia-Delclaux S Nottin R Stéphan F Use of prothrombin complex concentrate for excessive bleeding after cardiac surgery. Interact Cardiovasc Thorac Surg. 2012;15(3):382–389. 22623627 10.1093/icvts/ivs224 PMC3422937

[14] Bruce D Nokes TJ Prothrombin complex concentrate (Beriplex P/N) in severe bleeding: experience in a large tertiary hospital. Crit Care. 2008;12(4):R105. 18706082 10.1186/cc6987 PMC2575594

[15] Davidson S State of the art—how I manage coagulopathy in cardiac surgery patients. Br J Haematol. 2014;164(6):779–789. 24450971 10.1111/bjh.12746

[16] Schick KS Fertmann JM Jauch KW Hoffmann JN Prothrombin complex concentrate in surgical patients: retrospective evaluation of vitamin K antagonist reversal and treatment of severe bleeding. Crit Care. 2009;13(6):R191. 19948037 10.1186/cc8186 PMC2811941

[17] Ortmann E Besser MW Sharples LD An exploratory cohort study comparing prothrombin complex concentrate and fresh frozen plasma for the treatment of coagulopathy after complex cardiac surgery. Anesth Analg. 2015;121(1):26–33. 25822921 10.1213/ANE.0000000000000689

[18] Kozek-Langenecker SA Ahmed AB Afshari A Management of severe perioperative bleeding: guidelines from the European Society of Anaesthesiology: First update 2016. Eur J Anaesthesiol. 2017;34(6):332–395. 28459785 10.1097/EJA.0000000000000630

[19] American Society of Anesthesiologists Task Force on Perioperative Blood Management. Practice guidelines for perioperative blood management: an updated report by the American Society of Anesthesiologists Task Force on Perioperative Blood Management. Anesthesiology. 2015;122(2):241–275. 25545654 10.1097/ALN.0000000000000463

[20] Rossaint R Bouillon B Cerny V The European guideline on management of major bleeding and coagulopathy following trauma: fourth edition. Crit Care. 2016;20(1):1–55. 27072503 10.1186/s13054-016-1265-x PMC4828865

[21] Klein AA Arnold P Bingham RM AAGBI guidelines: the use of blood components and their alternatives 2016. Anaesthesia. 2016;71(7):829–842. 27062274 10.1111/anae.13489

[22] Rebers S Aaronson NK van Leeuwen FE Schmidt MK Exceptions to the rule of informed consent for research with an intervention. BMC Med Ethics. 2016;17:9. 26852412 10.1186/s12910-016-0092-6 PMC4744424

[23] Pabinger I Brenner B Kalina U; Beriplex P/N Anticoagulation Reversal Study Group. Prothrombin complex concentrate (Beriplex P/N) for emergency anticoagulation reversal: a prospective multinational clinical trial. J Thromb Haemost. 2008;6(4):622–631. 18208533 10.1111/j.1538-7836.2008.02904.x

[24] Lorenz R Kienast J Otto U Efficacy and safety of a prothrombin complex concentrate with two virus-inactivation steps in patients with severe liver damage. Eur J Gastroenterol Hepatol. 2003;15(1):15–20. 12544689 10.1097/00042737-200301000-00004

[25] Garvin S Muehlschlegel JD Perry TE Postoperative activity, but not preoperative activity, of antithrombin is associated with major adverse cardiac events after coronary artery bypass graft surgery. Anesth Analg. 2010;111(4):862–869. 19820236 10.1213/ANE.0b013e3181b7908c PMC3010354

[26] Rafiq S Johansson PI Ostrowski SR Stissing T Steinbrüchel DA Hypercoagulability in patients undergoing coronary artery bypass grafting: prevalence, patient characteristics and postoperative outcome. Eur J Cardiothorac Surg. 2012;41(3):550–555. 22011771 10.1093/ejcts/ezr001

[27] Russo A Grigioni F Avierinos JF Thromboembolic complications after surgical correction of mitral regurgitation incidence, predictors, and clinical implications. J Am Coll Cardiol. 2008;51(12):1203–1211. 18355659 10.1016/j.jacc.2007.10.058

[28] Weissman C Pulmonary complications after cardiac surgery. Semin Cardiothorac Vasc Anesth. 2004;8(3):185–211. 15375480 10.1177/108925320400800303

[29] Staudinger T Frass M Rintelen C Influence of prothrombin complex concentrates on plasma coagulation in critically ill patients. Intensive Care Med. 1999;25(10):1105–1110. 10551966 10.1007/s001340051019 PMC7094948

[30] Sarode R Milling TJ Jr Refaai MA Efficacy and safety of a 4-factor prothrombin complex concentrate in patients on vitamin K antagonists presenting with major bleeding: a randomized, plasma-controlled, phase IIIb study. Circulation. 2013;128(11):1234–1243. 23935011 10.1161/CIRCULATIONAHA.113.002283 PMC6701181

[31] Christensen MC Krapf S Kempel A von Heymann C Costs of excessive postoperative hemorrhage in cardiac surgery. J Thorac Cardiovasc Surg. 2009;138(3):687–693. 19698857 10.1016/j.jtcvs.2009.02.021

[32] Coakley M Hall JE Evans C Assessment of thrombin generation measured before and after cardiopulmonary bypass surgery and its association with postoperative bleeding. J Thromb Haemost. 2011;9(2):282–292. 21091865 10.1111/j.1538-7836.2010.04146.x

[33] Solomon C Rahe-Meyer N Sorensen B Fibrin formation is more impaired than thrombin generation and platelets immediately following cardiac surgery. Thromb Res. 2011;128(3):277–282. 21429567 10.1016/j.thromres.2011.02.022

[34] Lance MD Ninivaggi M Schols SE Perioperative dilutional coagulopathy treated with fresh frozen plasma and fibrinogen concentrate: a prospective randomized intervention trial. Vox Sang. 2012;103(1):25–34. 22211833 10.1111/j.1423-0410.2011.01575.x

[35] Gorlinger K Dirkmann D Hanke AA First-line therapy with coagulation factor concentrates combined with point-of-care coagulation testing is associated with decreased allogeneic blood transfusion in cardiovascular surgery: a retrospective, single-center cohort study. Anesthesiology. 2011;115(6):1179–1191. 21970887 10.1097/ALN.0b013e31823497dd

[36] Gorlinger K Fries D Dirkmann D Weber CF Hanke AA Schöchl H Reduction of fresh frozen plasma requirements by perioperative point-of-care coagulation management with early calculated goal-directed therapy. Transfus Med Hemother. 2012;39(2):104–113. 22670128 10.1159/000337186 PMC3364099

[37] Schochl H Maegele M Solomon C Görlinger K Voelckel W Early and individualized goal-directed therapy for trauma-induced coagulopathy. Scand J Trauma Resusc Emerg Med. 2012;20:15. 22364525 10.1186/1757-7241-20-15 PMC3306198

[38] Ostermann H Haertel S Knaub S Kalina U Jung K Pabinger I Pharmacokinetics of Beriplex P/N prothrombin complex concentrate in healthy volunteers. Thromb Haemost. 2007;98(4):790–797. 17938803

[39] Joint UKBTS/HPA Professional Advisory Committee. Shelf-life of frozen plasma components following thawing. http://www.transfusionguidelines.org. Published 2015. Accessed July 2017.

[40] Allen GA Wolberg AS Oliver JA Hoffman M Roberts HR Monroe DM Impact of procoagulant concentration on rate, peak and total thrombin generation in a model system. J Thromb Haemost. 2004;2(3):402–413. 15009455 10.1111/j.1538-7933.2003.00617.x

[41] Butenas S, van’t Veer C Mann KG “Normal” thrombin generation. Blood. 1999;94(7):2169–2178. 10498586

[42] Wolberg AS Monroe DM Roberts HR Hoffman M Elevated prothrombin results in clots with an altered fiber structure: a possible mechanism of the increased thrombotic risk. Blood. 2003;101(8):3008–3013. 12506014 10.1182/blood-2002-08-2527

[43] Franklin SW Szlam F Fernandez JD Leong T Tanaka KA Guzzetta NA Optimizing thrombin generation with 4-factor prothrombin complex concentrates in neonatal plasma after cardiopulmonary bypass. Anesth Analg. 2016;122(4):935–942. 26599794 10.1213/ANE.0000000000001098

[44] Percy CL Hartmann R Jones RM Correcting thrombin generation ex vivo using different haemostatic agents following cardiac surgery requiring the use of cardiopulmonary bypass. Blood Coagul Fibrinolysis. 2015;26(4):357–367. 25928274 10.1097/MBC.0000000000000243 PMC4888920

[45] Grottke O Braunschweig T Spronk HM Increasing concentrations of prothrombin complex concentrate induce disseminated intravascular coagulation in a pig model of coagulopathy with blunt liver injury. Blood. 2011;118(7):1943–1951. 21670472 10.1182/blood-2011-03-343046

[46] Schöchl H Voelckel W Maegele M Kirchmair L Schlimp CJ Endogenous thrombin potential following hemostatic therapy with 4-factor prothrombin complex concentrate: a 7-day observational study of trauma patients. Crit Care. 2014;18: R147. 25008277 10.1186/cc13982 PMC4227066

[47] Hanke AA Joch C Gorlinger K Long-term safety and efficacy of a pasteurized nanofiltrated prothrombin complex concentrate (Beriplex P/N): a pharmacovigilance study. Br J Anaesth. 2013;110:764–772. 23335567 10.1093/bja/aes501 PMC7094476

[48] Close V Purohit M Tanos M Hunter S Should patients post-cardiac surgery be given low molecular weight heparin for deep vein thrombosis prophylaxis? Interact Cardiovasc Thorac Surg. 2006;5(5):624–629. 17670663 10.1510/icvts.2006.137703

[49] Lauzier F Cook D Griffith L Upton J Crowther M Fresh frozen plasma transfusion in critically ill patients. Crit Care Med. 2007;35(7):1655–1659. 17522577 10.1097/01.CCM.0000269370.59214.97

[50] Myles PS Smith JA Forbes A Tranexamic acid in patients undergoing coronary-artery surgery. N Engl J Med. 2017;376(2):136–148. 27774838 10.1056/NEJMoa1606424

