# Supplementary material for: Retrospective Review of a Prothrombin Complex Concentrate (Beriplex P/N) for the Management of Perioperative Bleeding Unrelated to Oral Anticoagulation
Source: Clin Appl Thromb Hemost. 2018 Feb 7;24(7):1159–69. doi: 10.1177/1076029617753537 (PMC6714747; doi:10.1177/1076029617753537)
Supplement: Supplemental Material, Probe_study_supplementary_information_20Jul2017 - Retrospective Review of a Prothrombin Complex Concentrate (Beriplex P/N) for the Management of Perioperative Bleeding Unrelated to Oral Anticoagulation [file Probe_study_supplementary_information_20Jul2017.pdf]

SUPPLEMENTARY INFORMATION

Supplementary Table 1. Hemostatic treatment administered to patients with thromboembolic events (in all seven cases, the thromboembolic events were considered as possibly related to study medication).

	Cohort	Surgical procedure(s)	Study treatment	Thrombo-embolic event	Hemostatic co-medication
**Patient 1***	Prophylaxis	Lumbar puncture for intrathecal chemotherapy for promyelocytic leukemia	PCC alone (Cumulative dose: 2750 IU)	Acute myocardial infarction	• Platelets (13 hrs before and 11 hrs after PCC)<br>• Fibrinogen (12 hrs after PCC)
**Patient 2**	Treatment	Mitral valve repair, pulmonary vein isolation, Cox-Maze procedure	PCC (1000 IU) and FFP	Lacunar infarction	• Vitamin K (oral)<br>• Desmopressin (intravenous)<br>• Cryoprecipitate<br>• Platelets (45 mins before and 210 mins after PCC)<br>• Red blood cells (360 mins and 405 mins after PCC)
**Patient 3**	Treatment	Aortic root replacement plus hemi arch replacement plus coronary artery bypass grafting	PCC (1500 IU) and FFP	Embolism (cerebral ischemic event)	• Vitamin K (oral)<br>• Desmopressin (intravenous)<br>• Cryoprecipitate<br>• Platelets (immediately before and 170 mins after PCC)
**Patient 4**	Treatment	Aortic root replacement	PCC (2000 IU) and FFP	Cerebral ischemia	• Vitamin K (oral)<br>• Desmopressin (intravenous)<br>• Cryoprecipitate<br>• Platelets (1 min before, and 61 and 240 mins after PCC)<br>• Red blood cells (34 and 45 mins after PCC)
**Patient 5**	Treatment	Aortic valve replacement plus coronary	PCC alone (1000 IU)	Cerebral infarction	• Vitamin K (oral)<br>• Desmopressin (intravenous)<br>• Cryoprecipitate

	Cohort	Surgical procedure(s)	Study treatment	Thrombo-embolic event	Hemostatic co-medication
		artery bypass grafting			• Platelets (38 mins before PCC)<br>• Red blood cells (4, 440 and 580 mins after PCC)
**Patient 6**	Treatment	Re-do sternotomy plus coronary artery bypass grafting	PCC alone (1000 IU)	Acute myocardial infarction	• Vitamin K (oral)<br>• Desmopressin (intravenous)<br>• Cryoprecipitate<br>• Platelets (2.5 hrs after PCC)<br>• Red blood cells (160, 40 and 10 mins before PCC)
**Patient 7**	Treatment	Re-do aortic valve replacement plus coronary artery bypass grafting	PCC (1000 IU) and FFP	Embolism (cerebral ischemic event)	• Vitamin K (oral)<br>• Desmopressin (intravenous)<br>• Cryoprecipitate<br>• Platelets (4 mins before and 50 mins after PCC)<br>• Red blood cells (115 mins before and 19 mins after PCC)

*Patient died and the cause of death was ‘relapsed acute promyelocytic leukemia and acute myocardial infarction’.
